# HOMEODOMAIN GLABROUS 4 promotes DNA damage-induced stem cell death in *Arabidopsis thaliana* roots

**DOI:** 10.1007/s10265-026-01741-0

**Published:** 2026-08-10

**Authors:** Toshiki Wada, Mihiro Nakajima, Naoki Takahashi

**Affiliations:** Department of Life Sciences, School of Agriculture, Meiji University, 1-1-1 Higashi-mita, Tama-ku, Kawasaki, Kanagawa 214-8571 Japan

**Keywords:** *Arabidopsis thaliana*, DNA damage, HOMEODOMAIN GLABROUS 4, Root, Stem cell death, SUPPRESSOR OF GAMMA RESPONSE 1

## Abstract

Maintenance of genomic integrity is essential for sustained cell proliferation in plant meristems. In *Arabidopsis thaliana*, the DNA damage response is controlled by the transcription factor SUPPRESSOR OF GAMMA RESPONSE 1 (SOG1), which regulates the expression of genes involved in DNA repair, cell cycle arrest, and stem cell death. However, the mechanisms underlying the correlation between DNA damage signaling and the regulation of stem cell death remain incompletely understood. In this study, we identified HOMEODOMAIN GLABROUS 4 (HDG4), a member of the homeodomain leucine zipper class IV transcription factor family, as a factor promoting DNA damage-induced stem cell death in the root meristem. Reporter analyses revealed that *HDG4* expression is strongly induced in the root meristem following DNA damage. Moreover, genetic analysis revealed that DNA damage-induced stem cell death was significantly suppressed in the *hdg4* mutant. The DNA damage-associated repression of auxin efflux carriers, *PIN-FORMED* (*PIN*), was partially alleviated in the *hdg4* mutant. Consistent with this observation, the reduction in the auxin response in the root tip after DNA damage was attenuated in the *hdg4* mutant. These results indicate that HDG4 contributes to the suppression of auxin transport toward the root tip during DNA damage response, and reveal a transcriptional mechanism linking genome integrity surveillance with hormonal regulation of stem cell death.

**Supplementary Information:**

The online version contains supplementary material available at https://doi.org/10.1007/s10265-026-01741-0.

## Introduction

Plants are continually exposed to genotoxic stress induced by both endogenous and environmental factors, including reactive oxygen species, ultraviolet radiation, and ionizing radiation (Berdy 1980; Cooke et al. 2003; Frohnmeyer et al. 2003). Because vascular plants undergo continuous post-embryonic organ formation throughout their lifespan, the maintenance of genome integrity is especially critical in proliferative tissues, such as the shoot and root apical meristems. To ensure genome stability, land plants activate a DNA damage response that coordinates DNA repair, cell cycle arrest, the early onset of endoreplication, and stem cell death under severe conditions (Adachi et al. 2011; Fulcher and Sablowski 2009; Wada et al. 2025; Yoshiyama et al. 2009). In *Arabidopsis thaliana* (L.) Heynh., the DNA damage response is largely controlled by the NAC-type transcription factor SUPPRESSOR OF GAMMA RESPONSE 1 (SOG1), which acts downstream of the phosphatidylinositol 3-kinase-related kinases ATAXIA TELANGIECTASIA MUTATED (ATM) and ATM/RAD3-RELATED (ATR) (Sjogren et al. 2015; Yoshiyama et al. 2013). Phosphorylation by ATM and ATR activates SOG1, thereby enabling it to orchestrate a broad range of transcriptional programs involved in DNA repair, cell cycle regulation, and stress responses (Bourbousse et al. 2018; Ogita et al. 2018). Genome-wide association studies have identified numerous SOG1 target genes and established SOG1 as a master regulator of gene expression in DNA damage responses (Bourbousse et al. 2018; Ogita et al. 2018).

A distinctive feature of the DNA damage response is the selective elimination of stem cells in the shoot and root apical meristems in *A. thaliana* (Fulcher and Sablowski 2009). This spatial specificity suggests that plants employ a controlled developmental surveillance mechanism to remove damaged stem cells from their meristematic niches. Genetic analyses have shown that DNA damage-induced stem cell death depends on ATM, ATR, and SOG1, and the subsequent niche reorganization enables the recovery of root growth following genotoxic stress (Fulcher and Sablowski 2009; Furukawa et al. 2010; Johnson et al. 2018). Therefore, selective stem cell elimination is considered a key strategy for maintaining long-term meristem function and genome integrity.

Recent studies have revealed an important role of hormonal regulation in shaping the spatial output of the DNA damage response in *A. thaliana* roots. DNA double-strand breaks (DSBs) stimulate cytokinin biosynthesis and signaling, which downregulates the expression of several *PIN-FORMED* (*PIN*) auxin efflux carriers, including *PIN1*, *PIN3*, and *PIN4* (Takahashi et al. 2021). These changes reduce downward auxin transport and auxin accumulation in the root meristematic zone. Such hormonal reprogramming contributes to meristem size reduction and stem cell death under conditions of DNA damage (Takahashi et al. 2021). In addition, the Aux/IAA transcriptional repressors IAA5 and IAA29, which repress auxin signaling, are directly induced by SOG1 and promote DNA damage-induced stem cell death locally around the root stem cell niche (Takahashi et al. 2025). These findings reflect the interaction of SOG1-dependent transcriptional programs with auxin signaling pathways, which regulate the developmental output of the DNA damage response. Despite these advances, the mechanisms linking the SOG1 transcriptional network to localized developmental outputs remain unclear. Although several SOG1 target genes involved in DNA repair and checkpoint regulation have been identified (Bourbousse et al. 2018; Ogita et al. 2018), little is known about the factors that connect DNA damage signaling to developmental patterning processes, including stem cell death or auxin transport regulation in the root meristem.

Class IV homeodomain leucine-zipper (HD-Zip IV) proteins constitute a plant-specific family of transcription factors, with certain members involved in epidermal differentiation and developmental patterning (Nakamura et al. 2006; Schrick et al. 2023). In *A. thaliana*, this family comprises 16 members, including regulators, such as ARABIDOPSIS THALIANA MERISTEM LAYER 1 (ATML1), PROTODERMAL FACTOR 2 (PDF2), and GLABRA2 (GL2) (Schrick et al. 2023). However, the biological functions of several HD-Zip IV members remain poorly understood; among these proteins, HOMEODOMAIN GLABROUS 4 (HDG4) does not induce obvious developmental defects when mutated under standard growth conditions, suggesting that its function becomes apparent only under specific environmental or stress conditions (Nakamura et al. 2006). However, the involvement of HDG4 in stress-induced developmental responses remains unknown.

In the present study, we have identified HDG4 as a previously unrecognized component of the DNA damage response in *A. thaliana*. We demonstrated that *HDG4* expression is induced by DNA damage in an SOG1-dependent manner, and SOG1 associates with the *HDG4* genomic region following genotoxic stress. Moreover, results revealed that loss of HDG4 function suppressed DNA damage-induced stem cell death and alleviated the repression of multiple *PIN* genes. Overall, these findings identify HDG4 as an SOG1-dependent transcriptional effector linking the DNA damage response to auxin transport regulation and stem cell elimination in the root meristem.

## Materials and methods

### Plant materials and growth conditions

*A. thaliana* (accession Col-0) was used in this study. *sog1-101* (Ogita et al. 2018), *pSOG1:SOG1-Myc* (Yoshiyama et al. 2013), *pPIN1:PIN1-GFP* (Feraru et al. 2008), and *DR5rev:GFP* (Benková et al. 2003) have been described previously. The *hdg4-1* (GABI_168F12) and *hdg4-2* (WiscDsLox382F6) were obtained from the Arabidopsis Biological Resource Center (ABRC). The *pPIN1:PIN1-GFP hdg4-1* and *DR5rev:GFP hdg4-1* were generated via genetic crossing. Seeds were sown on Murashige and Skoog (MS) plates containing half-strength MS salts, 1% sucrose, 0.5 g/L 2-(N-morpholino) ethanesulfonic acid, and 1.2% phyto agar (pH 6.3), followed by incubation at 4 °C for 2 days. Subsequently, plates were placed vertically, and seedlings were grown under continuous light at 23 °C. For zeocin or hydroxyurea (HU) treatment, five-day-old seedlings were transferred to MS plates supplemented with zeocin (Thermo Fisher Scientific) or HU (Tokyo Chemical Industry Co., Ltd.) and grown vertically under continuous light conditions. To generate *promoter:GFP* fusion construct, the 2-kb promoter region upstream of the *HDG4* start codon was PCR-amplified from *A. thaliana* genomic DNA using the primer sets listed in Table S1 and cloned into the pDONR221 entry vector (Invitrogen) via a BP recombination reaction according to the instructions provided by the manufacturer. Next, the entry clone was introduced into the destination vector pGWB604 through LR recombination to generate GFP fusion constructs (Nakagawa et al. 2007). To generate the overexpression construct, the full-length open reading frame of *HDG4* was amplified from *A. thaliana* cDNA by PCR using the primer sets listed in Table S1, and cloned into the pDONR221 entry vector (Thermo Fisher Scientific) by a BP recombination reaction. The LR reaction was performed using the destination vector pGWB602 (Nakamura et al. 2010). The construct was introduced into *Agrobacterium tumefaciens* strain GV3101, which was used to generate stable transgenic plants using the floral dip method (Clough and Bent 1998).

### Quantitative real-time PCR (qRT–PCR)

Total RNA was extracted from *A. thaliana* roots using a Plant Total RNA Mini Kit (Favorgen). First-strand cDNA was synthesized from total RNA using ReverTra Ace qPCR RT Master Mix (Toyobo) according to the manufacturer’s instructions. qRT**-**PCR was performed using a KAPA SYBR Fast qPCR Kit (Kapa Biosystems) with 100 nM gene-specific primers, first-strand cDNAs, and primers listed in Table S1. PCR reactions were conducted using the PikoReal Real-Time PCR system (Thermo Fisher Scientific) under the following conditions: 95 °C for 5 min, followed by 50 cycles of denaturation at 95 °C for 10 s, annealing at 55 °C for 15 s, and extension at 72 °C for 30 s. Gene expression was analyzed using the 2^−∆∆Ct^ method (Livak and Schmittgen 2001) and normalized using *ACTIN2* as a reference gene. Three biological replicates were analyzed.

### Root growth analysis

Seedlings were grown vertically, and the position of each root tip was marked on the plate at 24 h intervals. Seedlings were photographed, and the distance between successive marks along the root axis was measured using the Fiji software (https://fiji.sc/) to quantify the root growth.

### Microscopic observation

Roots were stained with 10 μM propidium iodide (PI) for 1 min at room temperature. The root tips were observed using a confocal laser scanning microscope (LSM880; Zeiss). The area of cell death was measured using the Fiji software by defining the region in which PI infiltrated the cells.

### Chromatin immunoprecipitation quantitative PCR (ChIP–qPCR)

Seeds of the *pSOG1:SOG1-MYC* transgenic lines were germinated in liquid MS medium and cultured at 23 °C under continuous light with gentle shaking at 50 rpm. After two weeks, whole seedlings were treated with or without 20 µM zeocin for 2 h and fixed in 1% formaldehyde for 15 min. Seedlings were ground, and chromatin was extracted. Chromatin DNA was sheared into fragments of 150–500 base pairs (bp) through sonication. The chromatin associated with the SOG1-Myc fusion protein was immunoprecipitated using an anti-Myc antibody (Millipore). Quantitative PCR was performed using the primer sets listed in Table S1 to measure the enrichment of specific genomic regions in immunoprecipitated chromatin. PCR amplification was carried out using the PikoReal Real-Time PCR System (Thermo Fisher Scientific) under the following conditions: 95 °C for 5 min, followed by 70 cycles of denaturation at 95 °C for 10 s, annealing at 60 °C for 15 s, and extension at 72 °C for 30 s. Enrichment was calculated using the 2^−∆∆Ct^ method (Livak and Schmittgen 2001). Three biological replicates were analyzed.

### Statistical analysis

Data are presented as means ± standard deviation (SD). Normality and homogeneity of variance were assessed using the Shapiro–Wilk test and Brown–Forsythe test, respectively. A *p* value < 0.05 was considered to indicate a significant deviation from the assumptions of normality or homogeneity of variance. When the assumptions of normality and homogeneity of variance were satisfied, differences between two groups were evaluated using Student’s *t*-test, whereas comparisons among more than two groups were analyzed by one-way ANOVA followed by Dunnett’s multiple comparison test or Tukey’s HSD test, as appropriate. When normality was satisfied but homogeneity of variance was not, Welch’s ANOVA followed by Games–Howell’s multiple comparison test was used. When normality was not satisfied, the Kruskal–Wallis test followed by Dunn’s multiple comparison test with Holm correction was applied. Differences were considered statistically significant at *p* < 0.05. The statistical test used for each experiment is indicated in the corresponding figure legends.

## Results

### *HDG4* is induced by DNA damage in an ATM–SOG1-dependent manner

Previous studies identified numerous genes whose expression is induced in an SOG1-dependent manner in response to DNA damage (Bourbousse et al. 2018; Ogita et al. 2018). Among these candidates, we focused on genes encoding transcription factors that may mediate downstream developmental responses to genotoxic stress. Notably, *HOMEODOMAIN GLABROUS 4* (*HDG4*), which encodes an HD-Zip transcription factor, was identified as a SOG1-dependent DNA damage-responsive gene. Analysis of previously published microarray datasets showed that *HDG4* expression increased approximately fourfold in wild-type (WT) seedlings after 2 h of treatment with zeocin, a radiomimetic agent that induces DNA double-strand breaks (DSBs), whereas no significant induction was observed in the *sog1-1* mutant (Fig. S1a). A similar SOG1-dependent induction was observed after treatment with bleomycin, another DSB-inducing agent (Fig. S1b). In contrast, hydroxyurea (HU), which primarily causes replication stress rather than DSBs, did not considerably induce *HDG4* expression under the tested conditions (Fig. S1c). Overall, these results suggest that *HDG4* is preferentially induced in response to DSB-type DNA damage in an SOG1-dependent manner.

SOG1 is activated by the upstream kinases, ATM and ATR, which respond to distinct types of DNA damage (Sjogren et al. 2015; Yoshiyama et al. 2013). ATM is primarily activated by DSBs, whereas ATR responds primarily to replication stress and single-stranded DNA lesions (Culligan et al. 2004; Garcia et al. 2003). To examine whether these kinases are involved in *HDG4* induction, we analyzed previously published microarray data from γ-irradiated *atm-2* and *atr-2* mutants (Culligan et al. 2006). In WT plants, γ-irradiation induced *HDG4* expression; this induction was largely abolished in the *atm-2* mutant but remained detectable in the *atr-2* mutant (Fig. S1d). These results indicate that *HDG4* induction depends primarily on the ATM pathway, which is consistent with the activation of the ATM–SOG1 signaling cascade by DSBs.

**Fig. 1:**
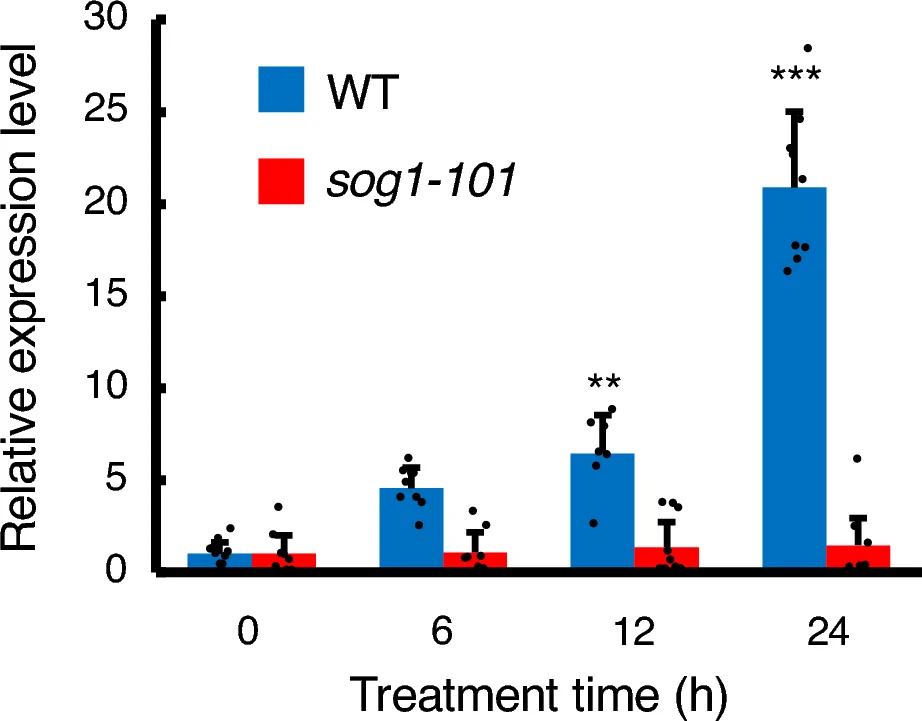
DNA damage induces *HDG4* expression in a SOG1-dependent manner. Transcript levels of *HDG4* in the presence of zeocin. Five-day-old WT and *sog1-101* seedlings were transferred onto MS plates supplemented with 10 µM zeocin and grown for 0, 6, 12, and 24 h. Total RNA was extracted from roots and subjected to qRT-PCR analysis. Transcript level of *HDG4* was normalized to that of *ACTIN2*, and were indicated as relative values, with that of the control set to 1. Data are presented as mean ± standard deviation (SD) calculated from three biological replicates. Significant differences from the 0 h control were determined by the Kruskal–Wallis test: **p < 0.01, ***p < 0.001

We next examined the temporal dynamics of *HDG4* expression following DNA damage by quantitative RT-PCR (qRT-PCR). Five-day-old WT seedlings were transferred to medium containing 10 μM zeocin, and total RNA was extracted from roots after 0, 6, 12, and 24 h of treatment. *HDG4* transcript levels increased approximately fivefold after 6 h of zeocin treatment compared to the untreated controls, and remained elevated thereafter (Fig. 1). In contrast, no clear induction of *HDG4* expression was detected in the *sog1-101* mutant after zeocin treatment (Fig. 1). These results indicate that *HDG4* is rapidly induced in response to DSBs in an SOG1-dependent manner.

### *HDG4* is induced predominantly in the root meristem in response to DNA damage

**Fig. 2:**
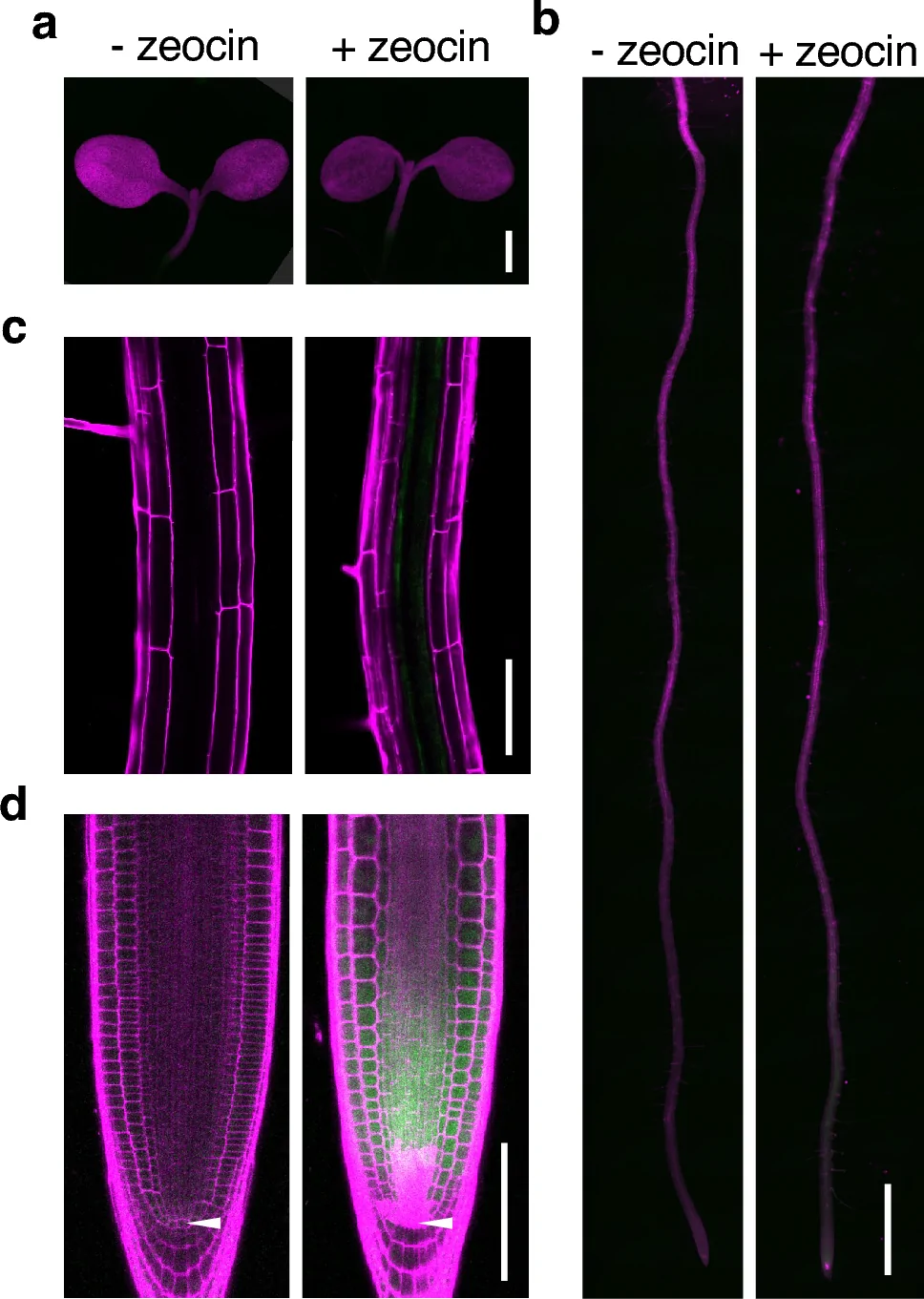
DNA damage induces *HDG4* in roots. **a**–**d** Five-day-old *pHDG4:GFP* seedlings were transferred onto MS plates supplemented with (+ zeocin) or without (− zeocin) 10 µM zeocin and grown for 18 h. GFP fluorescence in cotyledons (**a**), whole roots (**b**), root vasculatures (**c**), and root tips (**d**) was observed after counterstaining with PI. Arrowheads indicate the QC cells. Bars = 1 mm (**a**, **b**) and 100 μm (**c**, **d**)

To investigate the spatial pattern of *HDG4* induction after DNA damage, we generated transgenic plants harboring a *pHDG4:GFP* reporter construct, in which *GFP* expression was driven by a 2-kb upstream promoter region of *HDG4*. Five-day-old seedlings were transferred to medium containing 10 μM zeocin, and GFP fluorescence was observed after 18 h of treatment. Under control conditions, no GFP fluorescence was detected in either the shoots or roots, indicating that basal *HDG4* promoter activity is very low (Fig. 2). In contrast, zeocin treatment induced clear GFP fluorescence in the root vascular tissues, with the most prominent signal detected in the root meristem (Fig. 2c, d). To examine whether *HDG4* was also induced in response to replication stress, seedlings were treated with 2 mM HU. In contrast to the zeocin treatment, HU did not induce detectable GFP fluorescence in the root tip (Fig. S2). These results indicate that *HDG4* induction is most prominent in the root meristem in response to DSBs.

### SOG1 associates with the *HDG4* locus in response to DNA damage

**Fig. 3:**
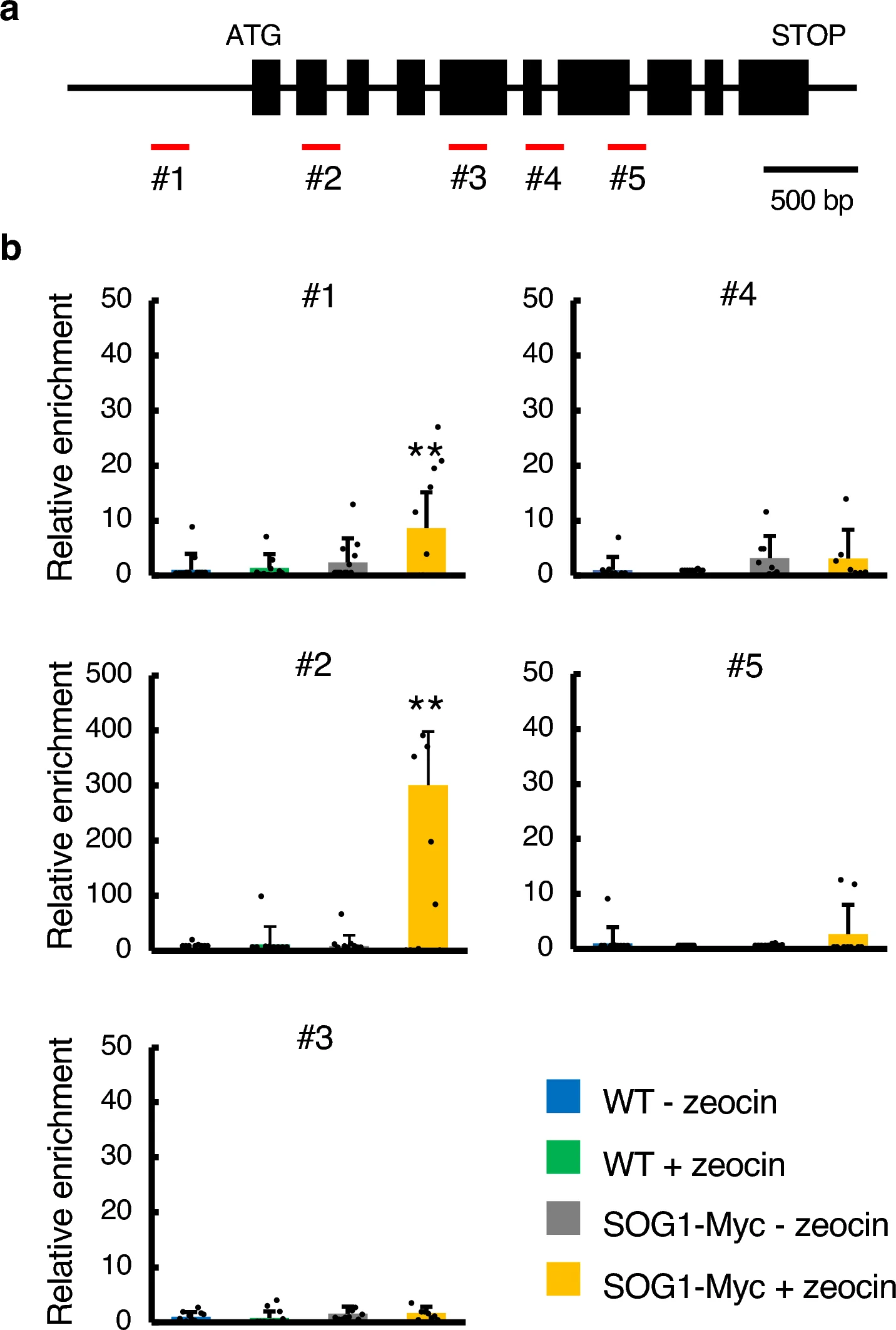
SOG1 directly binds to the *HDG4* locus. **a** Schematic representation of the *HDG4* locus. Black boxes and red lines indicate the coding regions and the sequences amplified by chromatin immunoprecipitation quantitative PCR (ChIP–qPCR), respectively. **b** Identification of SOG1-binding sites. Two-week-old WT and *pSOG1:SOG1-Myc* (SOG1-Myc) seedlings grown in liquid MS medium were cultured in a medium supplemented with (+ zeocin) or without (− zeocin) 20 µM zeocin for 2 h. WT was used as negative control. Genomic DNA was extracted from whole seedlings, and chromatin bound to SOG1-Myc was immunoprecipitated. qPCR was conducted to amplify the *HDG4* genomic regions shown in **a**. Fold enrichments of DNA fragments by ChIP–qPCR are indicated as relative values, with that for the WT control (− zeocin) set to 1. Data are presented as mean ± SD calculated from three biological replicates. For the data of SOG1-Myc, significant differences from the WT were determined by the Kruskal–Wallis test: **p < 0.01

DNA damage induces *HDG4* in an SOG1-dependent manner; hence, we investigated whether SOG1 directly associates with the *HDG4* locus via chromatin immunoprecipitation (ChIP) assays using transgenic plants expressing functional SOG1–Myc under the control of its native promoter (*pSOG1:SOG1-Myc*). ChIP-enriched DNA was quantified by qPCR using primer sets distributed across the *HDG4* locus, including both promoter and coding regions. Under normal growth conditions, no significant SOG1 binding was detected in any of the tested regions (Fig. 3). In contrast, after treatment with 20 μM zeocin for 2 h, SOG1 binding was detected at the *HDG4* locus. The strongest enrichment was observed in region #2, located around the second exon (Fig. 3), indicating that the association between SOG1 and *HDG4* locus is enhanced by DNA damage. SOG1 recognizes the consensus binding motif CTT(N)_7_AAG (Bourbousse et al. 2018; Ogita et al. 2018). Consistent with the prominent ChIP enrichment around region #2, we identified a motif-like sequence, GTTTAGAAAGAAG, located across the second exon and second intron, 227 bp downstream of the *HDG4* start codon in this region, further supporting the hypothesis that SOG1 associates with the *HDG4* locus in response to DNA damage. Overall, these results suggest that *HDG4* is directly regulated by SOG1 upon DNA damage.

### DNA damage-induced stem cell death is suppressed in *hdg4* mutants

**Fig. 4:**
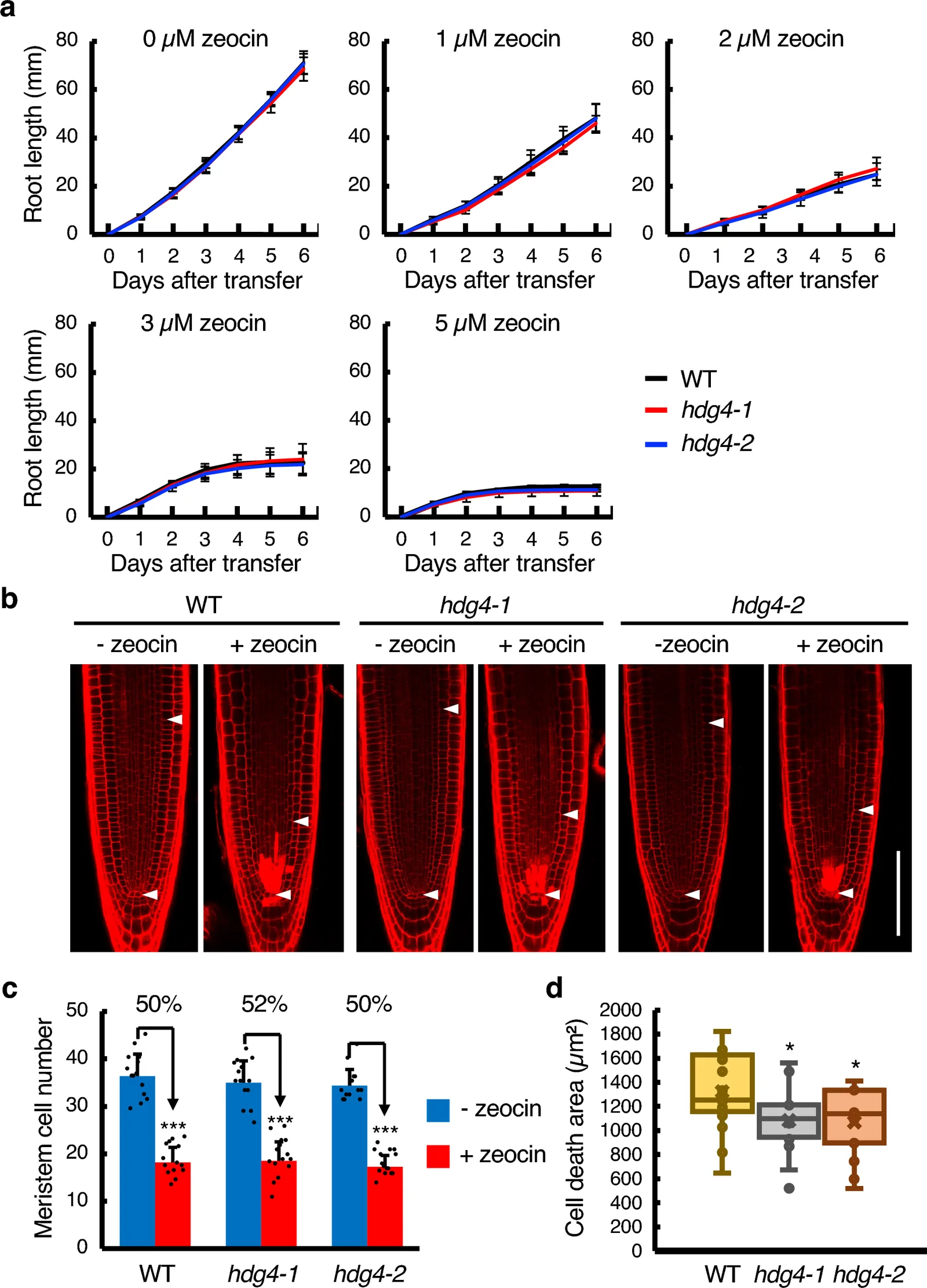
DNA damage-induced stem cell death is suppressed in *hdg4* mutants. **a** Root growth in *hdg4* mutants. Five-day-old WT, *hdg4-1*, and *hdg4-2* seedlings were transferred to MS plates supplemented with 0, 1, 2, 3, or 5 µM zeocin, respectively, and root length was measured every 24 h. Data are presented as mean ± SD (*n* > 8). Root length did not significantly differ among WT, *hdg4-1*, and *hdg4-2* (*p* > 0.05, Dunnett’s test). **b** Root meristem after zeocin treatment. Five-day-old WT, *hdg4-1*, and *hdg4-2* seedlings were transferred to MS plates supplemented with or without 10 μM zeocin, and grown for 18 h. Root tips were stained with PI and observed using a confocal laser scanning microscope. Arrowheads indicate the QC (lower) and the boundary between the meristematic zone and the transition zone (upper). Bar = 100 μm. **c** The number of cortical cells between the QC and the first elongated cell was counted. Data are presented as mean ± SD (*n* > 14). Significant differences from the control were determined by Student’s *t*-test: ***p < 0.001. **d** The area of PI-stained dead cells in roots grown in the presence of zeocin was measured (*n* > 13). In the box plots, center lines represent the medians; box limits indicate the 25th and 75th percentiles; whiskers extend to 1.5 times the interquartile range, and circles indicate each value in the data set. Significant differences from the WT were determined by Dunnett’s test: *p < 0.05

To investigate the physiological role of HDG4 in DNA damage responses, we characterized two independent *hdg4* mutant alleles. The *hdg4-1* (GABI_168F12) and *hdg4-2* (WiscDsLox382F6) lines harbor T-DNA insertions in the second exon and fifth intron of *HDG4*, respectively (Fig. S3a). qRT-PCR confirmed the absence of detectable *HDG4* transcripts in both lines (Fig. S3b), indicating the loss-of-function mutations. First, we examined whether the loss of *HDG4* affects the overall root growth under DNA damage conditions. Five-day-old WT and *hdg4* seedlings grown on MS medium were transferred to medium supplemented with 0, 1, 2, 3, or 5 µM zeocin and grown for an additional six days. Under both control and zeocin-treated conditions, the root growth of the *hdg4* mutants was indistinguishable from that of the WT (Fig. 4a), suggesting that HDG4 is not essential for overall root growth under these conditions. To further assess meristem activity, we measured root meristem size by counting the number of cortical cells between the quiescent center (QC) and the first elongated cell. In the WT roots, zeocin treatment for 18 h reduced meristem cell numbers by 49%, reflecting the DNA damage-induced suppression of cell proliferation (Fig. 4b, c). A similar reduction in the *hdg4* mutants (Fig. 4b, c) indicated that HDG4 is not a key regulator of the reduction in meristem size under these conditions.

We next examined DNA damage-induced stem cell death. Roots were treated with 10 µM zeocin for 18 h and stained with propidium iodide (PI) to visualize dead cells. In WT roots, cell death was predominantly detected in vascular initials and their daughter cells (Fig. 4b). Although a similar spatial pattern of stem cell death was observed in *hdg4* mutants, the extent of stem cell death, quantified based on intracellular PI staining in the root tip, was significantly reduced to approximately 80% of the WT level (Fig. 4b, d). These results suggest that HDG4 contributes to the maximal induction of stem cell death in response to DNA damage.

To next determine whether the reduced stem cell death observed in *hdg4* mutants affects root growth recovery after DNA damage, seedlings were treated with zeocin and subsequently transferred to zeocin-free medium. Root growth recovered similarly in *hdg4* mutants and WT seedlings during the recovery period (Fig. S4). These results suggest that reduced stem cell death in *hdg4* mutants does not significantly affect root growth recovery under the conditions tested.

### HDG4 contributes to DNA damage-induced suppression of auxin signaling

**Fig. 5:**
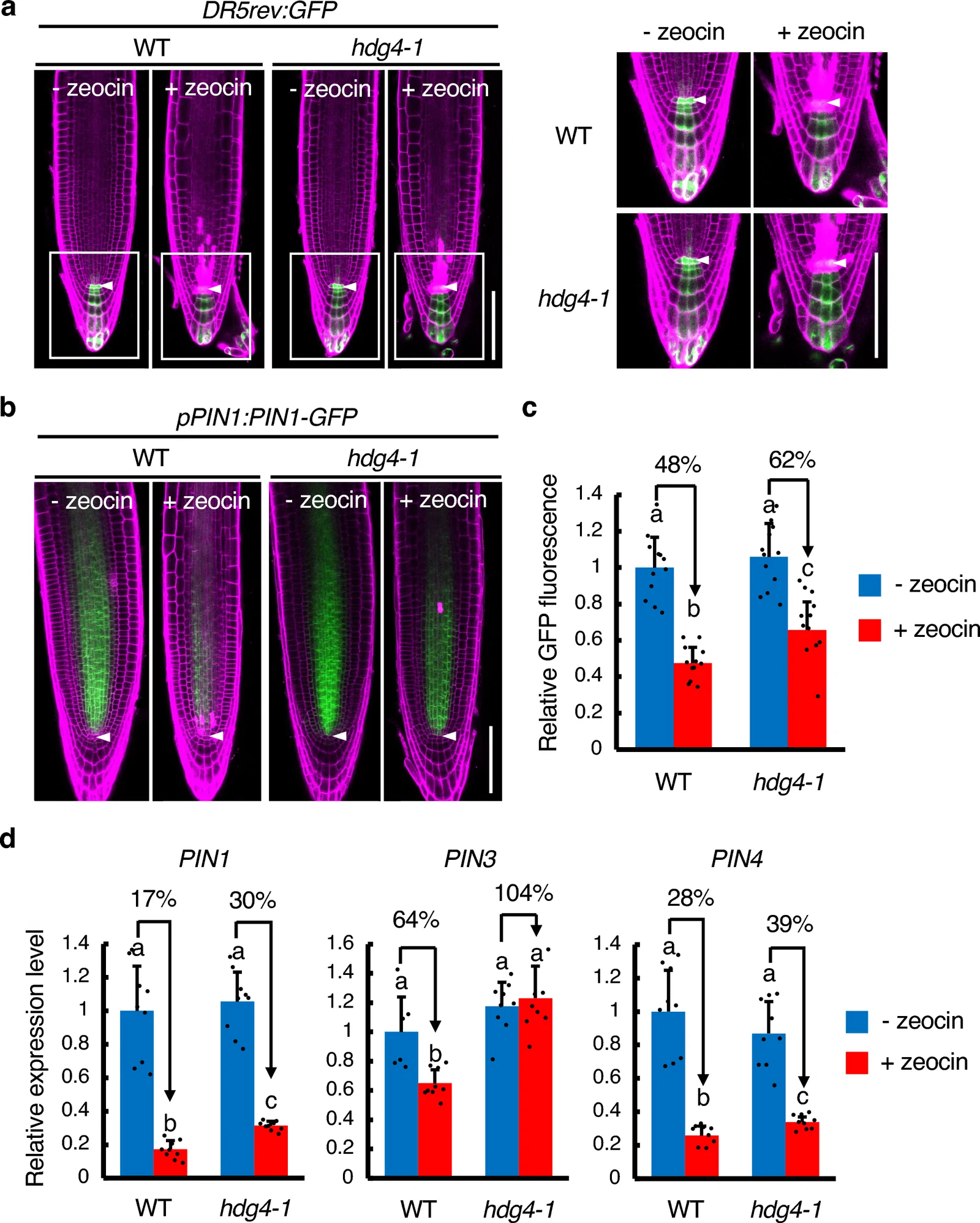
HDG4 is involved in DNA damage-induced suppression of auxin signaling. **a** Five-day-old WT and *hdg4-1* seedlings harboring *DR5rev:GFP* were transferred to MS plates supplemented with (+ zeocin) or without (− zeocin) 10 μM zeocin, and grown for 24 h. Roots were stained with PI and observed using a confocal laser scanning microscope. Magnified images of the areas marked by white boxes in *DR5rev:GFP* are shown on the right. **b** PIN1-GFP in the *hdg4* mutant under DNA damage conditions. Five-day-old seedlings of WT and *hdg4-1* harboring *pPIN1:PIN1-GFP* were treated with (+ zeocin) or without (− zeocin) 10 µM zeocin for 9 h. GFP fluorescence was observed after counterstaining with PI. Arrowheads indicate the QC cells; bar = 100 µm (**a** and **b**). **c** GFP fluorescence intensity of *pPIN1:PIN1-GFP* roots. GFP fluorescence in the root meristem is indicated as relative values, with that of the control without zeocin treatment set to 1. Data are presented as mean ± SD (*n* > 12). Different letters indicate significant differences (*p* < 0.05, Tukey’s HSD test). **d **Transcript levels of *PIN1*, *PIN3*, and *PIN4*. Five-day-old WT and *hdg4-1* seedlings were transferred to MS plates with (+ zeocin) or without (− zeocin) 10 µM zeocin, and grown for 9 h. Total RNA was extracted from the roots and subjected to qRT-PCR. Transcript levels of *PIN1*, *PIN3*, and *PIN4* were normalized to those of *ACTIN2*, and are indicated as relative values, with that for WT control set to 1. Data are presented as mean ± SD calculated from three biological and technical replicates. Different letters indicate significant differences (*p* < 0.05; Welch’s ANOVA)

Previous studies have shown that DNA damage reduces auxin signaling in the root apex (Takahashi et al. 2021). Upon DNA damage, the expression levels of several *PIN* auxin efflux carrier genes, including *PIN1*, *PIN3*, and *PIN4*, are downregulated, resulting in reduced auxin accumulation and signaling in the root tip. This reduction in auxin signaling contributes to the induction of stem cell death. Since DNA damage-induced stem cell death was suppressed in the *hdg4* mutants, we examined whether HDG4 contributes to the suppression of auxin signaling following DNA damage. Hence, we analyzed the auxin-responsive reporter *DR5rev:GFP* in the *hdg4-1* background. Seedlings were treated with zeocin, and GFP fluorescence in the root tips was investigated. Under control conditions, no significant difference in GFP fluorescence was observed between WT and *hdg4-1* roots. In contrast, under DNA damage conditions, GFP fluorescence was reduced in the WT roots, whereas this reduction was partially suppressed in the *hdg4-1* mutant (Fig. 5a). These results indicate that the DNA damage-induced reduction in the auxin response is partially alleviated in *hdg4-1*.

Next, we examined whether HDG4 affects *PIN1* expression under DNA damage conditions. The PIN1 protein levels were analyzed using the *pPIN1:PIN1-GFP* reporter line. In WT roots, zeocin treatment resulted in approximately 45% reduction in PIN1-GFP fluorescence (Fig. 5b, c). In contrast, the reduction in PIN1-GFP levels was considerably attenuated in the *hdg4-1* mutant, indicating that the repression of PIN1 expression under DNA damage is partially compromised in *hdg4*. In agreement with these results, qRT-PCR analysis revealed that DNA damage-induced downregulation of *PIN1*, *PIN3*, and *PIN4* transcripts was attenuated in *hdg4-1* compared to that in the WT (Fig. 5d). Taken together, these results suggest that HDG4 contributes to the DNA damage-induced repression of *PIN* gene expression, potentially contributing to reduced auxin signaling in the root tip.

## Discussion

Continuous cell proliferation in plant meristems underpins postembryonic organ development, and stability of this process requires the preservation of genome integrity. In *A. thaliana*, the DNA damage response is regulated through the ATM/ATR–SOG1 signaling pathway, which activates the transcriptional programs involved in DNA repair, cell cycle arrest, and stem cell death (Ogita et al. 2018; Yoshiyama et al. 2013). In both root and shoot apical meristems, DNA damage triggers the selective elimination of stem cells and their immediate daughter cells. This process is considered to protect meristem integrity while permitting subsequent regeneration of the stem cell niche (Fulcher and Sablowski 2009; Johnson et al. 2018). In this study, we identified HDG4 as an SOG1-dependent factor involved in the DNA damage response, which contributes to DNA damage-induced stem cell death. Our results further suggest that HDG4 contributes to the downregulation of *PIN* expression and the associated inhibition of auxin signaling under DNA damage. Together, these findings suggest that HDG4 functions as a downstream regulatory factor linking the inhibition of auxin transport to stem cell elimination associated with the SOG1-dependent DNA damage response. Thus, this study provides insights into a previously unrecognized transcriptional module that connects hormonal regulation, development, and genotoxic stress.

Genetic analyses have indicated that HDG4 preferentially contributes to the regulation of stem cell death during the DNA damage response. In the *hdg4* mutant, zeocin treatment caused meristem size reduction and root growth inhibition comparable to those in WT, whereas stem cell death was significantly suppressed. These observations suggest that HDG4 does not regulate all physiological responses induced by DNA damage, but specifically regulates stem cell elimination. SOG1 regulates distinct responses through multiple downstream modules, some of which are involved in DNA repair and cell cycle regulation (Ogita et al. 2018; Yoshiyama et al. 2009). For example, ANAC044 and ANAC085 are required for DNA damage-induced G2 arrest (Takahashi et al. 2019). Our findings extend this framework by identifying HDG4 as a downstream transcriptional regulator that contributes to stem cell death. HDG4 belongs to the HD-Zip IV family of transcription factors, which have been studied primarily in the context of epidermal differentiation and outer layer development (Chew et al. 2013; Nakamura et al. 2006; Schrick et al. 2023). Many HD-Zip IV mutants exhibit weak or no obvious phenotypes under normal growth conditions, suggesting functional redundancies or context-dependent roles (Nakamura et al. 2006). Our results show that HDG4 has a detectable function under genotoxic stress conditions, suggesting that HD-Zip IV transcription factors may contribute not only to developmental patterning but also to stress-responsive developmental regulation. In contrary, *HDG4* overexpression did not cause detectable changes in root growth or meristem size (Fig. S5). This observation suggests that HDG4 alone is not sufficient to trigger these developmental responses and may require additional DNA damage-induced factors or specific cellular contexts for its function.

Our results also implicate HDG4 in modulation of auxin signaling upon DNA damage. Previously, DNA damage was reported to induce cytokinin accumulation in the root transition zone, leading to the repression of auxin transporters, such as PIN1, PIN3, and PIN4, consequently reducing auxin transport toward the root tip (Takahashi et al. 2021). Hormonal reorganization contributes to stem cell death. In the *hdg4* mutant, the DNA damage-associated repression of *PIN1*, *PIN3*, and *PIN4* was partially alleviated, suggesting that HDG4 contributes to the pathway linking SOG1 signaling to reduced auxin transport. However, the molecular mechanisms underlying the effect of HDG4 on *PIN* expression remain unclear. This correlation may be attributable to HDG4-mediated direct or indirect regulation of auxin transport-associated genes; HDG4, belonging to the HD-Zip IV family, binds to specific cis-elements and influences their transcription in a context-dependent manner. Alternatively, HDG4 may act within the cytokinin–auxin regulatory circuit that reorganizes hormone distribution after DNA damage. In this scenario, HDG4 can potentially strengthen cytokinin-dependent transcriptional responses or facilitate the repression of *PIN* genes downstream of cytokinin signaling. Furthermore, HDG4 may modify the cellular state of the root meristem, thereby indirectly affecting hormone-related gene expression. These mechanisms potentially suggest that HDG4 acts as a mediator linking SOG1-dependent DNA damage signaling with hormone-regulatory networks that regulate auxin distribution. Further studies, including the identification of HDG4 target genes, are required to clarify the molecular basis of the HDG4-dependent regulation of auxin transport.

The partial suppression of stem cell death in the *hdg4* mutant suggests that HDG4 is not the sole regulator of this process. DNA damage-induced stem cell death is controlled by multiple mechanisms. For instance, *IAA5* and *IAA29* are directly induced by SOG1 in the stem cell region, which promote stem cell death through the local repression of auxin signaling (Takahashi et al. 2025). Therefore, HDG4 may function concurrently with these modules to reinforce the suppression of PIN-dependent auxin transport or to suppress auxin signaling at the root tip. These findings support a model in which SOG1 orchestrates a multilayered regulatory network that integrates hormonal signaling with local transcriptional responses to ensure the selective elimination of damaged stem cells.

In conclusion, our results identified HDG4 as an SOG1-dependent transcriptional regulator that contributes to the repression of auxin transport and stem cell death in the root meristem. Moreover, a new connection between the DNA damage response and hormonal control of DNA damage-induced stem cell death has been elucidated, which provides valuable insights into how genome surveillance mechanisms are integrated with developmental regulation to maintain meristem integrity.

## Supplementary Information

Below is the link to the electronic supplementary material.

Supplementary file1 (PDF 980 kb)

## Data Availability

The Arabidopsis Genome Initiative (AGI) numbers for SOG1, HDG4, PIN1, PIN3, and PIN4 are AT1G25580, AT4G17710, AT1G73590, AT1G70940, and AT2G01420, respectively. Data supporting the findings of this study are available from the corresponding author upon request.
