# Supplementary material for: HOMEODOMAIN GLABROUS 4 promotes DNA damage-induced stem cell death in Arabidopsis thaliana roots
Source: J Plant Res. 2026 Aug 10;139(5):885–95. doi: 10.1007/s10265-026-01741-0 (PMC13588868; doi:10.1007/s10265-026-01741-0)
Supplement: Supplementary file 1 [file 10265_2026_1741_MOESM1_ESM.pdf]

Electronic supplementary materials

Title:

HOMEODOMAIN GLABROUS 4 promotes DNA damage-induced stem cell death in *Arabidopsis thaliana* roots

Authors:

Toshiki Wada[1], Mihiro Nakajima[1], Naoki Takahashi[1]

Affiliation:

[1]Department of Life Sciences, School of Agriculture, Meiji University, Tama-ku, Kawasaki, Kanagawa 214-8571, Japan.

Journal:

Journal of Plant Research

Corresponding author

Naoki Takahashi

Department of Life Sciences, School of Agriculture, Meiji University

1-1-1 Higashi-mita, Tama-ku, Kawasaki, Kanagawa 214-8571, Japan

Tel.: +81-44-934-7036; Fax +81-44-934-7036

E-mail: takahashi@meiji.ac.jp

Contents:

Table S1

Fig. S1-S5

Table S1. Primers used in cloning, ChIP–qPCR, and qRT–PCR

Cloning

HDG4 promoter	5'-GGGGACAAGTTTGTACAAAAAAGCAGGCTTCTAACAAAGTTATCATAATCATAG-3'<br>5'-GGGGACCACTTTGTACAAGAAAGCTGGGTCTCCTTTTTCTTTCTTGTCTTTCG-3'
HDG4 CDS	5'-GGGGACAAGTTTGTACAAAAAAGCAGGCTTCATGGAAACGAAAGACAAGAAAG-3'<br>5'-GGGGACCACTTTGTACAAGAAAGCTGGGTCTTAATTACCGACGTCGTTGACG-3'

ChIP–qPCR

HDG4 #1	5'-GGACTCTCTGCTTCCTTCGAG-3'<br>5'-AGGAAGTGTAAACGGCGTCC-3'
HDG4 #2	5'-ACTGCAATCGAACAAGAGCC-3'<br>5'-TGTAAACTAAAAGGGCAAGAAGACG-3'
HDG4 #3	5'-TTGCGGAAGAAGAAAAGGCG-3'<br>5'-TCCTCATTCAGACACACACTCTC-3'
HDG4 #4	5'-AGCGCCAAAACGGCTCAGATC-3'<br>5'-AAAAGAGAGACAAGATTCAGTGACC-3'
HDG4 #5	5'-CTGAACGGTGGCTATCTGTG-3'<br>5'-CATTGGTTGTGGAAGTTTGAGG-3'

qRT–PCR

HDG4	5'-GGAAACGAAAGACAAGAAAGAAAAAG-3'<br>5'-ATCGGGAAATCTACAACCATCCAT-3'
PIN1	5'-CTCTCCACGTACTGGTTGTCGTTAC-3'<br>5'-CGGAAGAGATCTTCACATGTTTGTG-3'
PIN3	5'-CTTGCTGGATGAGCTACAGCTTTGG-3'<br>5'-CAAGTCAAACAGCCATGACGCCAAG-3'

PIN4	5’-CGAAAGAGTAATGCTAGAGGTGGTG-3’<br>5’-AATATCAGTCGTGTCATCACACTTG-3’
ACTIN2	5’-CTGGATCGGTGGTTCCATTC-3’<br>5’-CCTGGACCTGCCTCATCATAC-3’

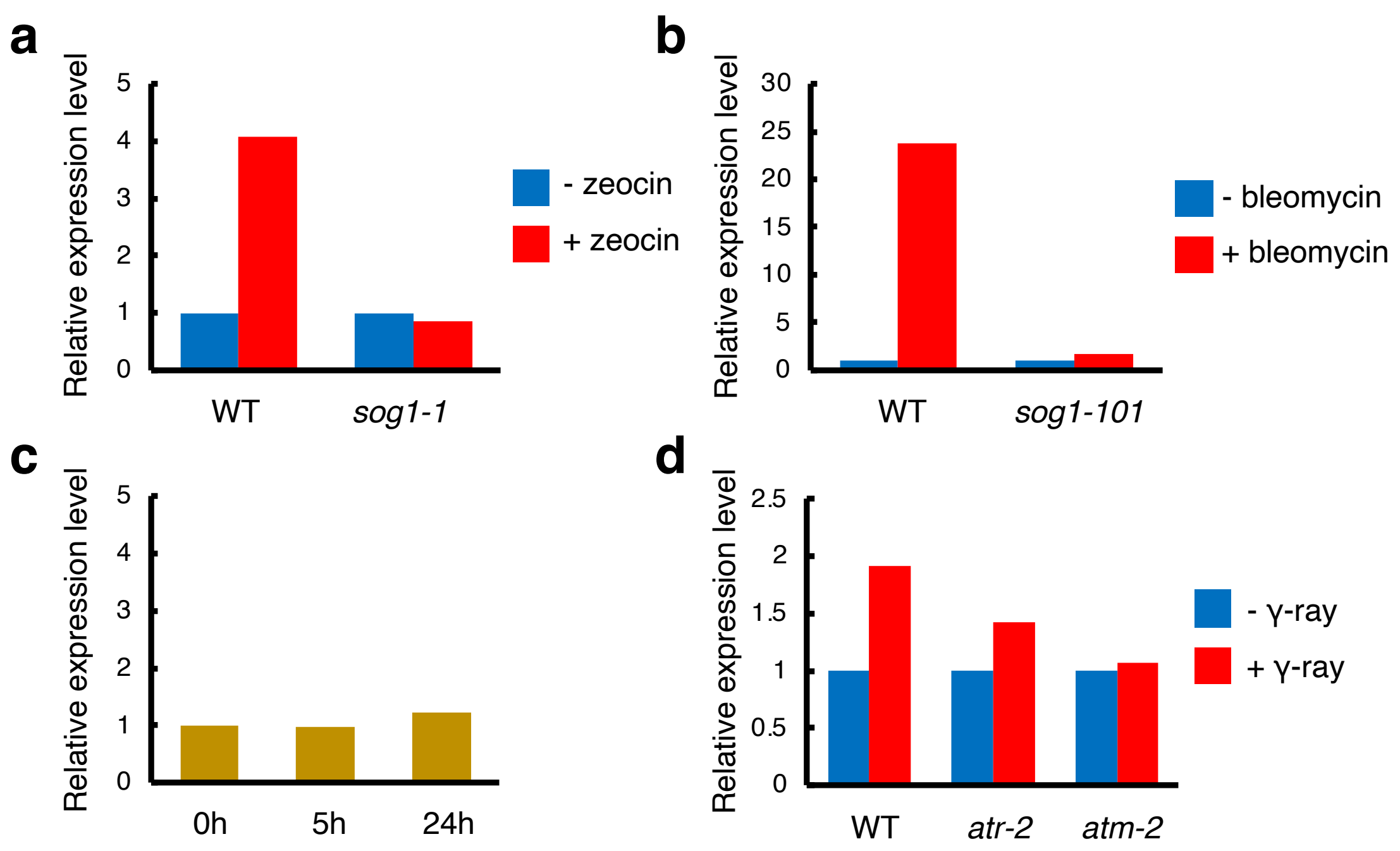

Fig. S1 DSBs induce *HDG4* in a SOG1-dependent manner

Transcriptional responses of *HDG4* to zeocin, bleomycin, hydroxyurea, and gamma irradiation. Data were obtained from the transcriptome results obtained by Ogita et al. (2018) (**a**), Takahashi et al. (2019) (**b**), Cools et al. (2010) (**c**), and Culligan et al. (2006) (**d**). (**a**) Five-day-old WT and *sog1-1* seedlings were treated with (+ zeocin) or without 15 μM zeocin (- zeocin) for 2 h, and total RNA was extracted from whole seedlings in three biological repeats. The relative transcript levels of *HDG4* are indicated with that of the control (- zeocin) set to 1. (**b**) Five-day-old WT and *sog1-101* seedlings were treated with (+ bleomycin) or without 0.6 μg/mL bleomycin (- bleomycin) for 10 h, and total RNA was extracted from root tips in three biological repeats. The relative transcript levels of *HDG4* are indicated compared with those of the control (- bleomycin) set to 1. (**c**) Seven-day-old WT seedlings were treated with 2 mM hydroxyurea for 0, 5 and 24 h, and the root tips were harvested for RNA extraction from two biological replicates. Relative transcript levels of *HDG4* are indicated compared with those of the 0 h control set to 1. (**d**) Five-day-old WT, *atr-2*, and *atm-2* seedlings were irradiated at 0 (- γ-ray) and 100 Gy gamma ray (+ γ-ray) and harvested 1.5 h after irradiation for total RNA isolation in two biological repeats. Relative transcript levels of *HDG4* compared with that of the control (- γ-ray) set to 1 are presented.

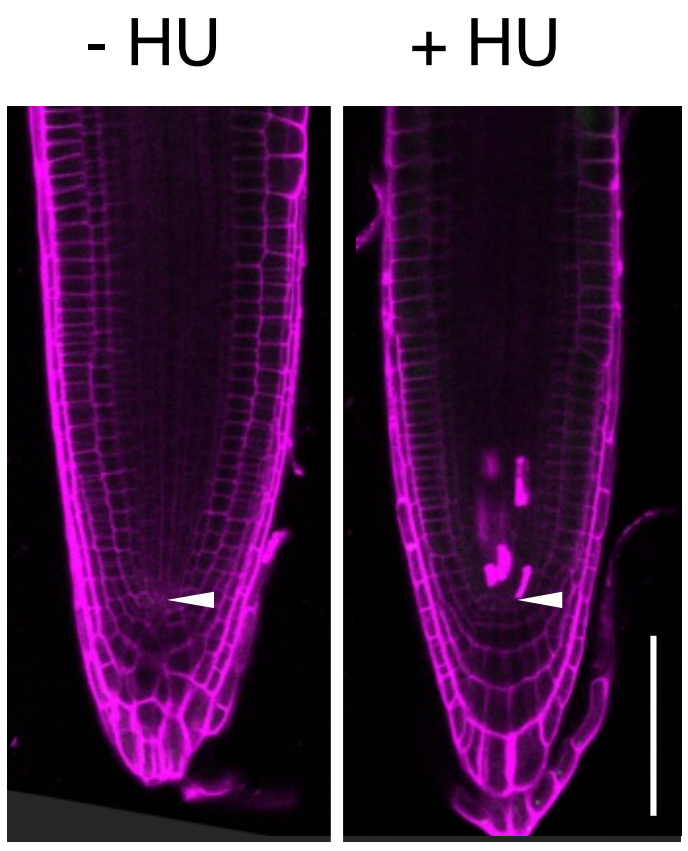

Fig. S2 *HDG4* expression under hydroxyurea treatment

Five-day-old *pHDG4:GFP* seedlings were transferred to MS plates supplemented with (+ HU) or without (- HU) 2 mM hydroxyurea and grown for 18 h. GFP fluorescence in the root tips was observed using a confocal laser scanning microscope after counterstaining with PI. Arrowheads indicate QC cells. Bars = 100 µm.

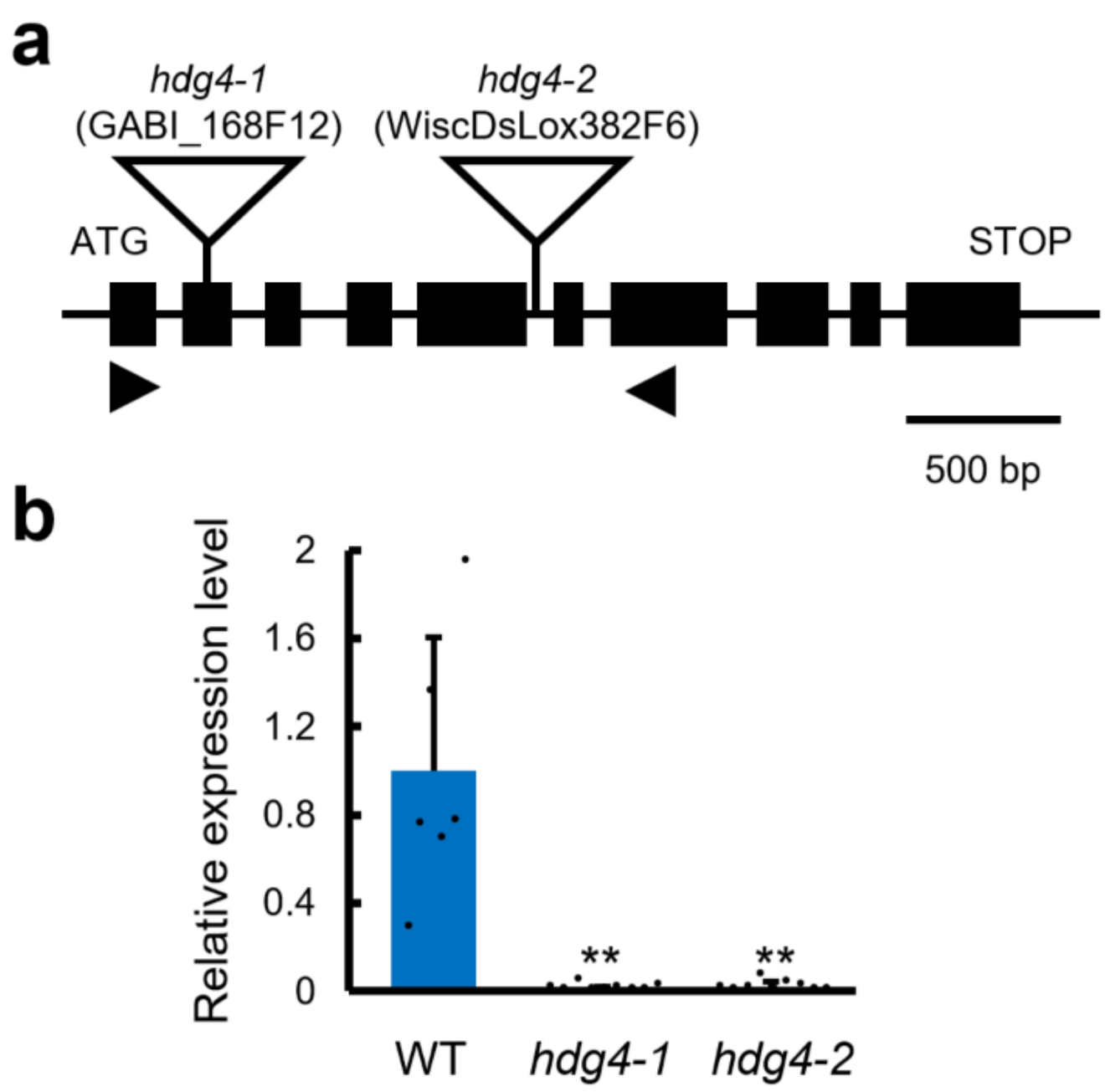

Fig. S3 Characterization of *hdg4* mutants

(**a**) Schematic representation of the *HDG4* gene. T-DNA insertion sites in *hdg4-1* (GABI_168F12) and *hdg4-2* (WiscDsLox382F6) are shown. Black boxes represent the exons. Arrowheads indicate the positions of primers used for qRT-PCR. (**b**) Differential levels of *HDG4* transcript. Total RNA was extracted from five-day-old WT, *hdg4-1*, and *hdg4-2* roots, and subjected to qRT-PCR. Transcript levels of *HDG4* were normalized to that of *ACTIN2*, and are indicated as relative values, with that for WT set to 1. Data are presented as mean ± SD calculated from three biological replicates. Significant differences from WT were determined by the Kruskal–Wallis test: $^{**}p < 0.01$.

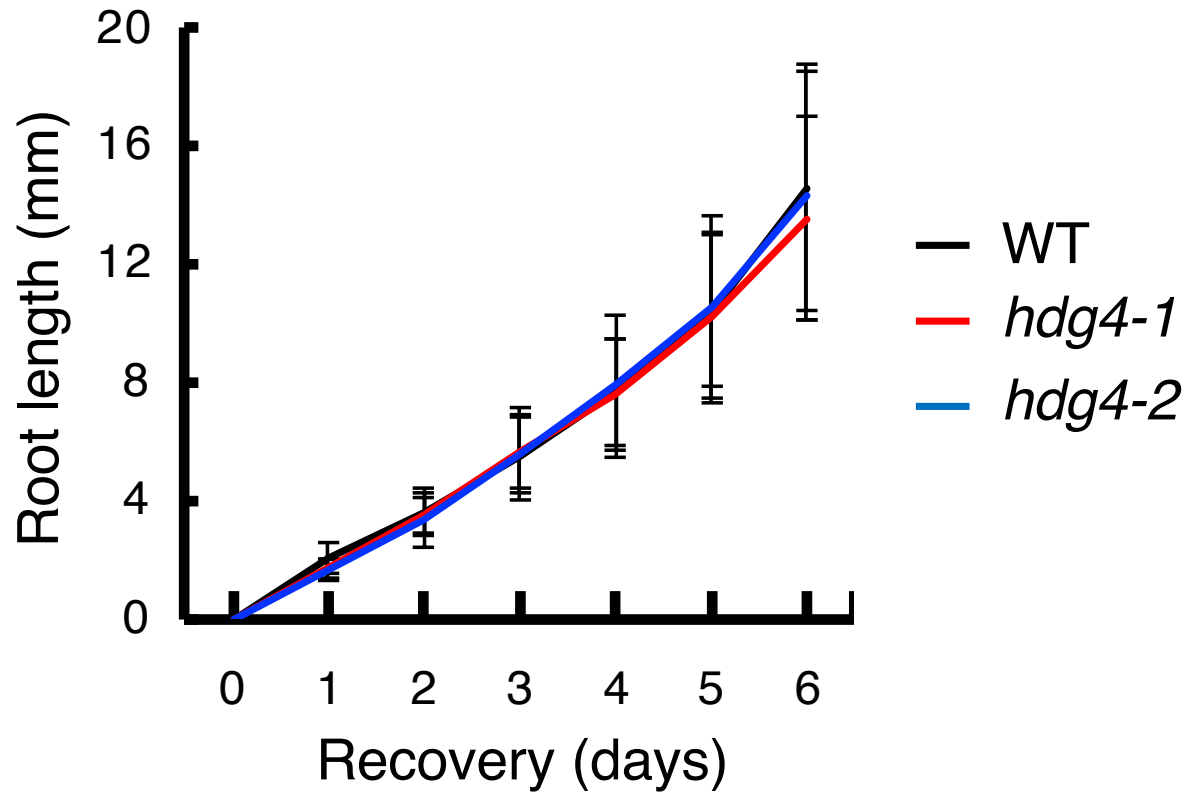

Fig. S4 Recovery of root growth in *hdg4* mutants from DNA stress
Five-day-old WT, *hdg4-1*, and *hdg4-2* seedlings were transferred onto an MS plate with 10 μM zeocin and grown for 18 h. The seedlings were then transferred back to a medium without zeocin, and root length was measured every 24 h. Data are presented as mean ± SD ($n > 12$). Root length did not significantly differ among WT, *hdg4-1*, and *hdg4-2* ($p > 0.05$, Dunnett's test).

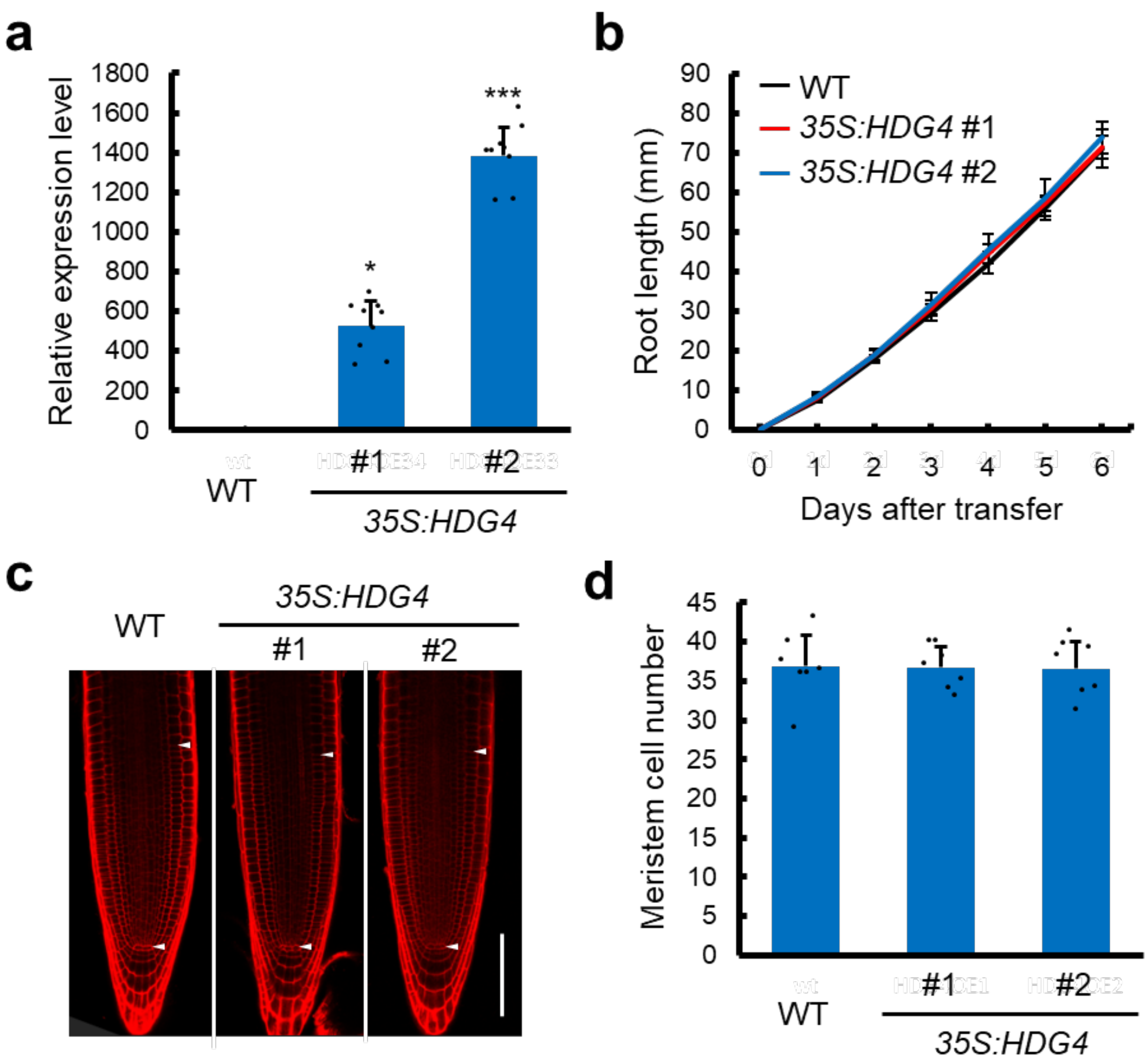

Fig. S5 Root growth in *HDG4*-overexpressing plants

(**a**) Transcript levels of *HDG4*. Total RNA was extracted from five-day-old WT and two independent *35S:HDG4* (#1 and #2) roots, and subjected to qRT-PCR. Transcript levels of *HDG4* were normalized to that of *ACTIN2*, and are indicated as relative values, with that for WT set to 1. Data are presented as mean ± SD calculated from three biological and technical replicates. Significant differences from WT were determined by the Kruskal–Wallis test: *$p < 0.05$, ***$p < 0.001$. (**b**) Root growth in *HDG4*-overexpressing plants. Five-day-old WT and two independent *35S:HDG4* plants (#1 and #2) were transferred to new MS plates, and root length was measured every 24 h. Data are presented as mean ± SD ($n > 8$). No significant differences were determined between WT and *HDG4*-overexpressing plants ($p > 0.05$, Dunnett's test). (**c**) Root meristem of *HDG4*-overexpressing plants. Root tips of five-day-old WT and *35S:HDG4* plants (#1 and #2) seedlings were observed after counterstaining with PI. Arrowheads indicate the QC (lower) and the boundary between the meristematic zone and the transition zone (upper). Bar = 100 μm (**d**) The number of cortical cells between the QC and the first elongated

cell was counted. Data are presented as mean ± SD ($n > 7$). Meristem cell number did not significantly differ among WT and *HDG4*-overexpressing plants ($p > 0.05$, Dunnett's test).
